# Patterns in Attribute Selection and Development Reporting in Patient Preference Studies Between 2007 and 2024: A Systematic Literature Review

**DOI:** 10.36469/001c.162119

**Published:** 2026-06-26

**Authors:** Siu Hing Lo, Rebekah Hall, Joy Wong, Gin Nie Chua

**Affiliations:** 1 Acaster Lloyd, London, UK; 2 Purdie Pascoe, London, UK

**Keywords:** patient preferences, discrete choice experiments, attribute selection, attribute development, patient and public involvement, research reporting, review

## Abstract

**Background:**

Discrete choice experiments (DCE) are commonly used for understanding patient preferences. However, their validity relies upon appropriate attribute selection and development. The present review aimed to identify reporting patterns and gaps to inform future reporting of patient preference studies.

**Methods:**

Ovid (MEDLINE and EMBASE) was used to search terms for “patients,” “discrete choice experiments,” and “attribute selection.” Two independent reviewers screened studies against PICOS eligibility criteria, focusing on formative attribute development methods and reporting quality in patient preference DCEs up to 2024. Only full-text journal publications detailing attribute selection and development in patient preference DCEs were included. A coding form was developed and used to capture reporting on formative research used for attribute selection or development. Data synthesis employed a narrative approach following PRISMA guidelines.

**Results:**

Six categories of formative methods were identified across 28 studies: patient qualitative concept elicitation (n = 27; 96%), literature reviews (n = 21; 75%), expert consultation (n = 21; 75%), quantitative prioritization (n = 17, 61%), quantitative pilot DCE surveys (n = 13; 46%), and qualitative cognitive debriefing ( n =7; 25%). Most studies stated objectives (>85%), but methodological transparency decreased substantially for other elements, including rationale for choice of method, sample characteristics, sampling methods, data collection procedures data analysis, and results for formative research methods involving primary data collection. Most studies reported attribute lists following formative research (>75% across methods), but across formative methods fewer than half overall reported how formative findings informed attribute selection, level selection, or wording decisions. While 96% of studies included patients as research participants, only 7% reported engaging patients as research partners.

**Conclusions:**

The findings revealed inconsistent reporting of formative methods for attribute selection and development. Reporting of formative method details and results was mixed, with particularly low levels of reporting for how formative research results informed attribute selection, level selection, and wording. Furthermore, few studies engaged patients as research partners, suggesting another key area for development in the field of patient preference research. Improved reporting standards are needed to support methodological clarity and strengthen the validity of patient preference research. Patient engagement in the development of studies may further strengthen the patient relevance of patient preference studies.

## INTRODUCTION

Discrete choice experiments (DCEs) have emerged as the most commonly used stated preference method for understanding stakeholder preferences in healthcare, particularly for eliciting patient preferences towards medical interventions.^1,2^ In a DCE survey, participants are presented with a series of choice tasks comparing hypothetical alternatives, each described by specific attributes such as efficacy, mode of administration, or side-effect probability for a study on patient preferences for treatments. Through systemic variation of these attributes across choice tasks, researchers can estimate the relative importance of different treatment characteristics to patient preferences.^2^

The validity and reliability of DCE findings depend critically on the appropriate selection and specification of attributes. These attributes must comprehensively capture the essential characteristics of relevant interventions while remaining interpretable to respondents.^3^ When relevant intervention characteristics are excluded or misunderstood, results may be compromised through attribute non-attendance or lack of external validity.^3,4^ Therefore, the process of attribute selection and development represents a foundational step in DCE design that directly influences the validity of results and their applicability to real-world healthcare decisions.

The importance of attribute selection and development is also reflected in recent regulatory guidances for patient preference studies. The final updated 2026 Food and Drug Administration (FDA) guidance on patient preference information expanded its previous guidance on attribute selection, framing and presentation of attributes.^5^ The draft International Council for Harmonisation of Technical Requirements for Pharmaceuticals for Human Use E22 guideline also emphasizes the importance of appropriate selection of attributes and attribute levels.^6^

Despite the importance of attribute selection, current reporting practices in DCE publications show considerable heterogeneity.^4,7–11^ While Helter and Boehler (2016)^7^ established a framework for attribute development, a recent review by Gonzalez Bohorquez et al (2024),^8^ examining high-quality qualitative methods used for the development of DCEs, highlighted that standardized reporting remains a challenge. Recent developments like the DIRECT Checklist^12^ for reporting DCEs in health and increased publication of separate attribute selection and development papers^4,9–11^ demonstrate growing recognition of the importance of transparent reporting. However, reporting practices of attribute selection and development in DCE studies have not been reviewed. By analyzing studies that provide descriptions of the formative research stages, this study aims to identify patterns and gaps in reporting practices that may inform the further development of enhanced reporting guidance for researchers. The present literature review included all DCE studies examining patient preferences for medical interventions which reported on study details relating to the methods and processes for DCE attribute selection and development.

## METHODS

### Search Strategy

Searches were conducted in Medline and Embase via Ovid, combining 3 main concepts: “patients” (eg, “patient,” “people living with”), “discrete choice experiment” (eg, “conjoint analysis,” “discrete choice experiment,” “DCE”), and “attribute selection” (eg, “attribute*adj3 select*,” “attribute* adj3 develop*”). The search terms included no date restrictions (**Table 1**).

**Table 1.** Search Terms

**Step**	**Search String**
1	(Patient or patients or people living with or people diagnosed with or people being treated or people with).tw.
2	(Conjoint or conjoint analysis or conjoint measurement or conjoint studies or conjoint choice experiment or part-worth utilities or functional measurement or paired comparisons or pairwise choices or discrete choice experiment or DCE or discrete choice modeling or discrete choice modelling or discrete choice conjoint experiment or stated preference).tw.
3	DCE-MRI.mp.
4	1 and 2
5	4 not 3
6	protocol.tw.
7	((attribute* adj3 select*) or (attribute* adj3 develop*)).tw.
8	(DCE adj3 develop*).tw.
9	6 or 7 or 8
10	5 and 9
11	remove duplicates from 10

### Eligibility Criteria

Eligibility criteria were developed using the PICOS framework where applicable.^13^ Studies were eligible for inclusion only if they met all specified criteria. For population criteria, studies must have focused on patient populations with any health condition requiring medical intervention. No restrictions were placed on participants’ age or ethnicity. Studies examining preferences of other stakeholders such as clinicians or caregivers were excluded.

All types of medical intervention were eligible for inclusion with no restriction on intervention type. As this was a methodological review examining reporting practices, comparator groups were not applicable.

Regarding outcomes, studies were required to report on the methods and processes of DCE attribute selection and development. This included reporting on at least one of the following: primary research conducted to select and develop DCE attributes, evidence sources consulted/literature review conducted for attribute selection, decision-making processes for attribute selection, or methods for finalizing attribute selection and development. Studies that listed methods or evidence sources used but did not further describe any methods or evidence sources were excluded.

For study design characteristics, only DCEs examining patient preferences for medical interventions were included. Studies were required to be full-text, peer-reviewed manuscript published in English. Conference abstracts and non-peer-reviewed publications were excluded.

This criteria framework was applied during both the initial screening of titles and abstracts and the subsequent full-text review. Any uncertainties about study eligibility were resolved through discussion between two reviewers, with arbitration by a third reviewer when necessary.

### Study Selection

Two reviewers (R.H. and J.W.) independently screened the first 10% (or minimum 200) of titles and abstracts against the eligibility criteria. Following assessment of inter-reviewer agreement, remaining references were screened by a single reviewer (J.W.) when agreement exceeded 80%. The same process was applied to full-text screening. Disagreements were resolved through discussion with a third reviewer (S.H.L.).

### Data Extraction and Coding

A standardized data extraction and coding form was developed in Microsoft Excel and piloted on a subset of included studies. Data extraction captured study characteristics (authors, publication year, objectives). A coding form was developed to capture any reporting (yes/no) on elements of formative research used to inform attribute selection and development by one reviewer (S.H.L.). The coding form was subsequently independently tested by two sets of two reviewers (S.H.L. and J.W., or G.N.C. and J.W.) for >20% of included studies (6 studies), with disagreements resolved through discussion.

For primary formative research methods, codes captured reporting on research objectives, rationale for choice of method, sample characteristics, sampling methods, data collection procedures, data analysis and results. For literature reviews, codes captured reporting on review objectives, rationale for choice of method, search terms and inclusion/exclusion criteria, database(s) used, screening results/flow chart, and review results.

For each method, it was also coded whether the authors reported an attribute list following completion of the formative research, as well as whether there was any reporting on how results of the formative research had informed attribute selection, attribute level selection and attribute wording.

Additionally, coding also captured any reporting (yes/no) on Patient and Public Involvement (PPI) in the research process or patient engagement as research partners rather than as study participants. Any reporting on PPI/patient engagement in studies was also extracted for a simple qualitative content analysis.

### Data Synthesis

Given the anticipated heterogeneity in reporting practices, we employed a narrative synthesis approach,^14^ primarily informed by a count analysis of codes supplemented by a qualitative review of the included studies. The narrative synthesis was structured around the type of formative method used for attribute selection and development, patterns in reporting of formative methods and results, and reporting on decision-making regarding how formative research results were used to inform attribute selection and development. The review follows PRISMA guidelines.^15^

## RESULTS

### Study Selection and Characteristics

**Figure 1.**
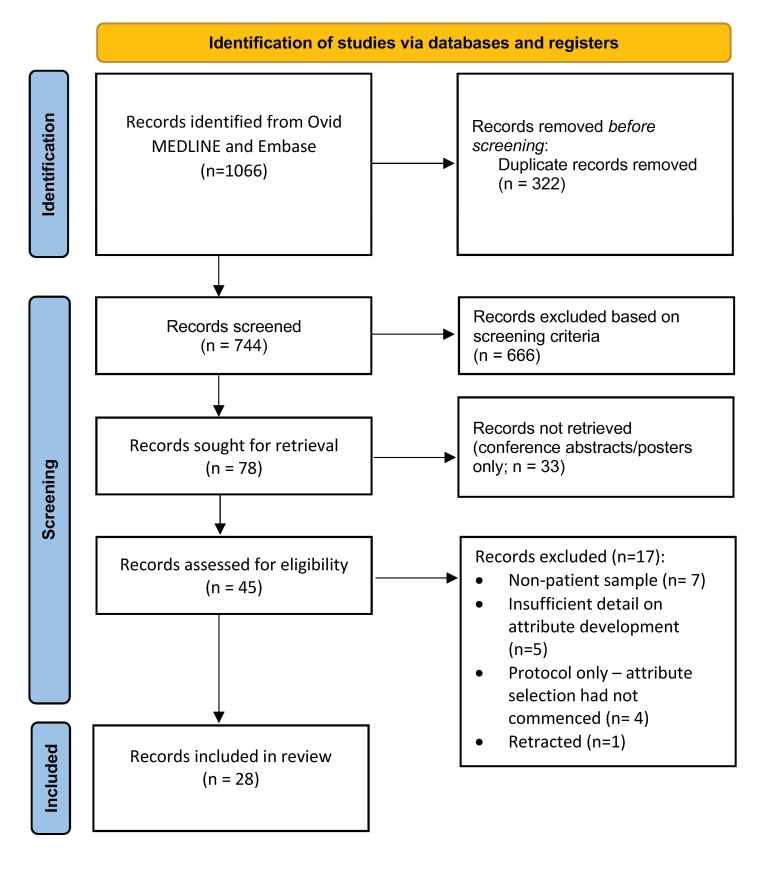
PRISMA Diagram of Article Screening

The initial search via Ovid MEDLINE identified 744 articles for title/abstract screening, from which 666 publications were excluded (**Figure 1**). Records were screened out if it was clear from the title/abstract that (1) the study was not a DCE; (2) the study population did not include patients; (3) “attribute selection,” “attribute development,” “DCE development,” or “protocol” were not mentioned in the title/abstract.

Seventy-eight records were sought for retrieval. Of the 78 records sought for retrieval, 33 were conference abstracts/posters for which a supplementary search did not identify an associated manuscript publication. Of the remaining 45 records, 17 full-text articles were excluded due to (1) non-patient sample (n = 7), (2) insufficient detail on attribute development (n = 5), (3) protocol only–attribute selection had not commenced (n = 4), or (4) retraction (n = 1). After screening, a total of 28 articles were included in the study.^10,16–42^

Articles (**Supplementary Table S1**) were published between 2007 and 2024, with a median publication year of 2020. Disease and intervention areas included oncology (n = 6; 21%), diabetes (n = 3; 11%), pregnancy (n = 3; 11%), arthritis (n = 3; 11%), digital technologies in healthcare (n = 2; 7%), as well as dermatology, osteoporosis, chronic non-cancer pain, hypodontia, lower back pain, mild cognitive impairment, HIV, coordinated care, dementia, chronic liver disease and depression (n = 1; 4% for each).

### Overview of Formative Attribute Selection and Development Methods

Six categories of formative methods for attribute selection and development were identified across the included studies. Patient qualitative concept elicitation was the most commonly used method (n = 27; 96%), followed by literature reviews (n = 21; 75%) and expert consultation (n = 21; 75%). Quantitative prioritization exercises were used by 17 studies (61%), quantitative pilot DCE survey by 13 studies (46%), and qualitative cognitive debriefing by 7 studies (25%).

Patient qualitative concept elicitation was conducted using various methods. Over half of the 27 studies used focus groups (n = 15/27; 56%), while approximately one-third employed semistructured interviews (n = 8/27; 30%). Additional methods included iterative interviews, interviews, analysis from interview transcripts and patient group discussion (n = 1/27; 4% for each). Ten studies (37%) used a combination of these qualitative methods.

Expert consultation methods varied across the 21 studies that employed this approach. Focus groups were most common (n = 6/21; 29%), followed by interviews (n = 4/21; 19%), and semistructured interviews (n = 3/21; 14%). Other methods included discussions (n = 2/21; 10%), as well as workshops, group and individual consultations, email consultations, iterative expert discussions, and Delphi methods (n = 1; 5% for each).

Quantitative prioritization exercises were used in 17 studies primarily through ranking exercises (n = 8; 47%), voting, combined ranking and Likert scales, Delphi methods, or combined ranking and rating (n = 2; 12%) for each. One study (6%) conducted ranking exercises within interviews or focus groups.

Statements on any PPI in the research process was reported in 6 (21%) of the included studies. Of these, only 2 (7%) described patient engagement as research partners and having engaged patients actively in study design decisions, beyond participation in qualitative research or survey completion. Patient engagement activities included involvement in the study design (including discussions on and reviewing of study materials for appropriateness and relevance) in general, as well as input on the final list of attributes and levels for the DCE.

### Reporting Across Formative Research Methods for Attribute Selection and Development

The reporting of study elements varied substantially across categories of formative research methods (**Table 2**). Research objectives were reported by over 75% of studies using each method, except for qualitative cognitive debriefing at 71% (n = 5/7). The rationale for choosing specific methods was rarely reported across any formative methods, with reporting rates consistently below 30% for all method types.

**Table 2.** Proportion of Papers Reporting Methods Aspects and Research Results by Formative Method

**Study Reporting Element**	**Literature Reviews (n=21), n (%)**	**Patient Qualitative Concept Elicitation (n=27), n (%)**	**Expert Consultation (n=21), n (%)**	**Quantitative Prioritization (n=17), n (%)**	**Qualitative Cognitive Debriefing (n=7), n (%)**	**Quantitative Pilot DCE Survey (n=13), n (%)**
Objectives	20 (95)	27 (100)	18 (86)	15 (88)	5 (71)	12 (92)
Rationale	2 (10)	3 (11)	3 (14)	1 (6)	2 (29)	0 (0)
Search terms and inclusion/ exclusion criteria	10 (48)	–	–	–	–	–
Database(s)	12 (57)	–	–	–	–	–
Screening results/ flow chart	7 (33)	–	–	–	–	–
Sample characteristics	–	23 (85)	16 (76)	12 (71)	6 (86)	11 (85)
Sampling methods	–	17 (63)	7 (33)	12 (71)	3 (43)	4 (31)
Data collection procedures	–	17 (63)	6 (29)	6 (35)	3 (43)	5 (38)
Analysis methods	–	18 (67)	8 (38)	11 (65)	0 (0)	5 (38)
Results	10 (48)	13 (48)	9 (43)	13 (76)	3 (43)	5 (38)

Literature reviews showed variable reporting across methodological aspects. Review objectives were reported by 95% of studies (n = 20/21). Other elements were reported less frequently: rationale for choice of method (n = 2/21; 10%), databases searched (n = 12/21; 57%), search terms and inclusion/exclusion criteria (n = 10; 48%), screening results/flow charts (n  = 7; 33%) and results of the review (n = 10; 48%).

Sample characteristics were reported by most studies using primary research methods, with reporting rates generally exceeding 75%. Quantitative prioritization was the exception with a reporting rate of sample characteristics of 71% (n = 12/17). Sampling methods were less consistently described across all primary research methods. Quantitative prioritization studies showed the highest rates of sampling methods reporting (n = 12/17; 71%), followed by patient qualitative concept elicitation (n = 17/27; 63%). Expert consultation, qualitative cognitive debriefing, and quantitative pilot DCE survey studies all had reporting rates of sampling methods at less than 50%.

Data collection procedures and analysis methods were reported by fewer studies across most primary research methods. Patient qualitative concept elicitation studies were most likely to describe their data collection procedures (n = 17/27; 63%) and their analysis methods (n = 18/27; 67%). While quantitative prioritization studies demonstrated similar rates for analysis methods (n = 11/17; 65%), they were less likely to report their data collection procedures (n = 6/17; 35%). Other methods showed substantially lower reporting rates for both data collection procedures and analysis methods: qualitative cognitive debriefing (n = 3/7; 43%, and 0%, respectively), quantitative pilot DCE survey (n = 5/13; 38% for each) and expert consultation (n = 6/21; 29% and n = 8/21; 38%, respectively).

The reporting of results from primary research methods varied by method type. Quantitative prioritization exercises showed the highest rates of results reporting (n = 13/17; 76%); followed by patient qualitative concept elicitation (n = 13/27 48%). Both expert consultation results (n = 9/21) and qualitative cognitive debriefing results (n = 3/7) were reported by 43% of studies, while quantitative pilot DCE survey results (n = 5/13) were reported by 38%.

### Reporting on Attribute Lists Following Completion of Formative Research

The majority of studies reported attribute lists following completion of their formative research methods. Patient qualitative concept elicitation studies most consistently provided attribute lists (n = 23/27; 89%) after completing the formative research, followed by literature reviews (n = 16/21; 81%). Quantitative prioritization exercises resulted in documented attribute lists in 10 of 17 studies (59%), while qualitative cognitive debriefing and quantitative pilot DCE surveys reported attribute lists in 4 of 7 studies (57%) and 4 of 13 studies (31%) respectively. However, where studies did not provide explicit attribute lists following completion of formative methods, changes to the original or draft attribute list were typically described in text format.

### Reporting on Attribute Selection and Development-Related Decision-Making

Reporting on how results of formative research methods informed decision-making regarding attribute selection and development are presented by method category in **Table 3**. Overall, few studies reported on decision-making regarding attribute selection and development, with reporting on how results of formative research informed decisions on attribute selection, attribute level selection and attribute wording being reported by less than half of the studies across most categories of formative research methods.

**Table 3.** Frequency of Studies Reporting How Formative Research Results Informed Attribute Selection, Attribute-Level Selection, and Attribute Wording by Formative Method

**Attribute Reporting Element**	**Literature Reviews (n=21), n (%)**	**Patient Qualitative Concept Elicitation (n=27), n (%)**	**Expert Consultation (n=21), n (%)**	**Quantitative Prioritization (n=17), n (%)**	**Qualitative Cognitive Debriefing (n=7), n (%)**	**Quantitative Pilot DCE Survey (n=13), n (%)**
Attribute selection	3 (14)	14 (52)	8 (38)	14 (82)	2 (29)	3 (23)
Attribute-level selection	4 (19)	13 (48)	7 (33)	0 (0)	2 (29)	4 (31)
Attribute wording	2 (10)	12 (44)	6 (29)	0 (0)	3 (43)	0 (0)

The main exception to the overall pattern of results were the 82% of studies using quantitative prioritization methods (n = 14/17) that described how results had informed attribute selection decisions. No quantitative prioritization results were reported to have informed attribute level selection or attribute wording.

Around half of studies using patient qualitative concept elicitation, a key formative method used to inform attribute selection and development, reported how the results informed attribute selection ( n = 14/27; 52%), attribute level selection (n = 13/27; 48%) and attribute wording (n = 12/27; 44%).

Less than half of studies using expert consultation reported how results informed attribute selection (n = 8/21; 38%), attribute level selection (n = 7/21; 33%) and attribute wording (n = 6/21; 29%).

For studies using qualitative cognitive debriefing of the draft DCE survey instrument, 43% reported how results informed attribute wording (n = 3/7), typically a key objective of cognitive debriefing. How cognitive debriefing results had informed attribute selection and attribute level selection (n = 2/7; 29% for each) was reported for around a quarter of studies.

Few studies including a quantitative pilot of the DCE survey reported on how the pilot results informed attribute selection and development. Attribute-level selection (n = 4/13; 31%) was most frequently reported to have informed, followed by attribute selection (n = 3/13; 23%).

Few studies including a literature review reported on how evidence from the review informed attribute selection (n = 3/21; 14%), attribute level selection (n = 4/21; 19%) or attribute wording (n = 2/21; 10%).

## DISCUSSION

This literature review examined reporting practices for attribute selection and development in patient DCEs. The included patient DCEs covered a wide range of therapeutic areas, including multiple studies in oncology, diabetes, gynecology/obstetrics, and arthritis. The findings suggest substantial variation in reporting across different formative research methods as well as different aspects of methods and results reporting, highlighting a relative lack of transparency in attribute selection and development in patient DCEs, even in publications providing a detailed description of DCE survey development.

Patient qualitative concept elicitation was the most commonly reported formative research method (96% of studies), signaling widespread recognition that patient preference studies need to present attributes in an appropriate choice context that are relevant to the intended target patient population. However, while nearly all studies included patients as participants, few (7%) described patient engagement in study design and development. In a recently published paper on the development of a patient preference DCE as informed by patient perspectives and advice from the FDA, the study authors detailed how feedback from research partners had altered and improved the study materials for formative qualitative research and educational material and illustrations used in the DCE, as well as the overall DCE study design, including sample inclusion criteria and planned subgroup analyses.^43^ The updated 2026 FDA guidance on patient preference information and draft ICH E22 guideline also highlight the value of patient input in the development of patient preference studies.^5,6^ The lack of engagement of patients as research partners in the vast majority of patient DCEs published before 2025 therefore represents another key area for development in the field of patient preference research.

Some consistent reporting patterns emerged across different categories of formative research. Research objectives were generally well-documented, but reporting decreased substantially for other aspects such as sampling, data collection procedures, analysis, and results of primary formative research. A key implication of lack of reporting in these areas is that the validity of the formative research cannot be assessed. If sampling details are unknown, it is unclear to what extent the sample was representative of the target patient population. If data collection procedures are not reported, it is not possible to assess whether the approach to research data could have introduced bias. If formative research results are not published, it is not possible to assess if the formative research provided adequate evidence for the final attribute selection and DCE survey instrument.

The present review also found that most studies reported attribute lists following formative research, suggesting recognition of the importance of documenting research outputs. However, the comparatively high rates of attribute list reporting compared to decision-making process reporting indicated that researchers may prioritize documenting what was decided over how and why decisions were made.

Formative research results reporting varied considerably by method type. Quantitative prioritization exercises showed highest reporting rates, likely reflecting the typical use of these methods to reduce the number of attributes in the final DCE and the ability to report results in a concise manner (eg, table with ranking results). In contrast, patient qualitative concept elicitation showed mixed results reporting, with around half of studies reporting results. This is despite concept elicitation among the target patient population being a core formative method to ensure patient-relevant attribute selection and development. Similarly, patient qualitative cognitive debriefing showed low reporting on results used to inform attribute wording, despite this typically being a primary objective of cognitive debriefing. Low levels of reporting on qualitative formative research results presents a critical gap and hinders the evaluation of the validity of the formative research and ultimately the relevance of patient DCE findings to real-world healthcare decisions. Low levels of results reporting were also observed for other categories of formative research, including literature reviews, expert consultation and quantitative pilot DCEs.

Incomprehensive reporting hinders the evaluation of formative research and ultimately limits the patient-centricity and informativeness of patient DCE studies to healthcare decision-making. The DIRECT Checklist provides valuable reporting guidance for DCEs and highlights the need to report on how attributes are derived.^12^ Recent regulatory guidelines also emphasize the importance of formative research and the appropriate selection of attributes and levels.^5,6^ However, our findings suggest that more detailed, practical reporting guidelines for attribute selection, development, and piloting procedures may be required to ensure consistent reporting.

Future DCE studies should prioritize transparent reporting for formative research, including method selection rationale, sampling methods, data collection procedures, data analysis, research results, and explicit documentation on how formative research findings inform attribute selection and development. Further, patient engagement as research partners represents an important opportunity for improving the patient centeredness of patient preference studies.

Several limitations should be considered when interpreting our findings. First, our literature search focused on DCE studies providing in-depth descriptions for the purpose of attribute selection and development. On the one hand, this may have introduced selection bias toward studies with higher-quality reporting, potentially underestimating the true extent of poor reporting in the broader patient preference literature. On the other hand, it may not have captured formative research for DCE studies that did not explicitly state informing attribute selection and development as an objective of the study. Second, our review assessed whether relatively broad aspects of formative research were reported on using a binary categorization (yes/no) rather than providing a qualitative assessment of the comprehensiveness of reporting on each of these aspects. As such, it is possible that studies that were classified as reporting on all aspects would still not have comprehensively reported on attribute selection and development. The results therefore do not provide a complete picture of underreporting on formative research for DCE attribute selection and development. Third, our review did not assess alignment between stated objectives for formative research and reporting on how such formative research informed decision-making relating to attribute selection, level selection and attribute wording. The review findings on the level of reporting on decision-making around attribute selection and development should therefore be interpreted as indicators of reporting patterns for patient DCEs up to 2024, and not a perfect assessment of reporting on decision-making for each individual included study.

## CONCLUSION

There are mixed levels of reporting on formative research for attribute selection and development in DCEs focused on patient preferences. While most studies clearly reported their objectives, crucial methodological details—such as rationale for method choice, sampling, data collection, analysis, formative research results, and integration of results in decision-making around attribute and level selection and wording—were inconsistently reported. These findings support the need for greater standardization and clearer reporting to enhance robustness and trust in patient preference research.

### Disclosures

The authors declare no conflicts of interest.

## Supplementary Material

Online Supplementary Material

## Data Availability

Available on request; only publicly available data were used for analysis.
