# Supplementary material for: Patterns in Attribute Selection and Development Reporting in Patient Preference Studies Between 2007 and 2024: A Systematic Literature Review
Source: J Health Econ Outcomes Res. 2026 Jun 26;13(1):280–7. doi: 10.36469/001c.162119 (PMC13264046; doi:10.36469/001c.162119)
Supplement: Online Supplementary Material [file jheor_2026_13_1_162119_351338.pdf]

Journal of Health Economics and Outcomes Research

Online Supplementary Material

Patterns in Attribute Selection and Development Reporting in Patient Preference Studies Between 2007-2024: A Systematic Literature Review. *JHEOR*. 2026;13(1):280-287. doi:10.36469/jheor.2026.162119

Table S1: List of Included Studies Detailing Formative Research Methods for Attribute Selection and Development

This supplementary material has been provided by the authors to give readers additional information about their work.

This is an open-access article distributed under the terms of the Creative Commons Attribution 4.0 International License (CCBY-4.0). View this license's legal deed at http://creativecommons.org/licenses/by/4.0 and legal code at http://creativecommons.org/licenses/by/4.0/legalcode for more information.

Table S1. List of Included Studies Detailing Formative Research Methods for Attribute Selection and Development

Title	Authors	Year	Objective	Citation	Condition/ Intervention
**Developing attributes and levels for discrete choice experiments using qualitative methods**	Joanna Coast, Sue Horrocks	2007	• To quantify patient preferences for access to dermatology specialist services alongside other potentially important aspects of the service.<br>• To provide a case study describing how attributes and their levels were developed for a study of access to dermatology specialist services for non-urgent skin conditions	Coast J, Horrocks S. Developing attributes and levels for discrete choice experiments using qualitative methods. *J Health Serv Res Policy*. 2007;12(1):25-30. doi:10.1258/135581907779497602. PMID: 17244394.	Dermatology
**Nominal group technique to select attributes for discrete choice experiments (DCEs): an example for drug treatment choice in osteoporosis**	Mickael Hiligsmann, Caroline van Durme, Piet Geusens, Benedict Gc Dellaert, Carmen D Dirksen, Trudy can der Weijden, Jean-Yves Reginster, Annelies Boonen	2013	To test the feasibility of using the nominal group technique (NGT) to select attributes for DCEs	Hiligsmann M, van Durme C, Geusens P, et al. Nominal group technique to select attributes for discrete choice experiments: an example for drug treatment choice in osteoporosis. *Patient Prefer Adher*. 2013;7:133-9. doi:10.2147/PPA.S38408. Epub 2013 Feb 7. PMID: 23412964; PMCID: PMC3572758.	Osteoporosis
**Return of incidental findings (IFs) in genomic medicine: measuring what patients value—development of an instrument to measure preferences for information from next-generation testing (IMPRINT)**	Caroline Savage Bennette, Susan Brown Trinidad, Stephanie M. Fullerton, Donald Patrick, Laura Amendola, Wylie Burke, Fuki M. Hisama, Gail, P. Jarvik, Dean A. Regier, David L. Veenstra	2013	• To identify, in the context of genetic testing for colon cancer susceptibility, the attributes and levels of IFs that are most important to, and cognitively understood by, patients<br>• To develop a DCE instrument that will enable the quantification of patients' personal utility for IFs from next-generation sequencing technologies	Bennette CS, Trinidad SB, Fullerton SM, et al. Return of incidental findings in genomic medicine: measuring what patients value—development of an instrument to measure preferences for information from next-generation testing (IMPRINT). *Genet Med.* 2013;15(11):873-881. doi:10.1038/gim.2013.63	Oncology
**Discrete choice experiment attribute selection using a multinational interview study: treatment features important to patients with type 2 diabetes mellitus**	Anna Rydén, Stephanie Chen, Emuella Flood, Beverly Romero, Susan Grandy	2017	To inform attribute and attribute-level selection in order to develop a DCE survey designed to examine preferences for GLP-1RA treatments among patients with type 2 diabetes	Rydén A, Chen S, Flood E, Romero B, Grandy S. Discrete choice experiment attribute selection using a multinational interview study: treatment features important to patients with type 2 diabetes mellitus. *Patient*. 2017;10(4):475-487. doi:10.1007/s40271-017-0225-0. PMID: 28315192.	Type 2 diabetes
**Qualitative development of a discrete choice experiment for physical activity interventions to improve knee osteoarthritis**	Daniel Pinto, Margaret K Danilovich, Paul Hansen, Daniel J Finn, Rowland W Chang, Jane L Holl, Aleen W Heinemann, Ulf Bockenholt	2017	To describe the qualitative process used to develop attributes and attribute levels for inclusion in a discrete choice experiments (DCE) for older adult physical activity interventions	Pinto D, Danilovich MK, Hansen P, et al. Qualitative development of a discrete choice experiment for physical activity interventions to improve knee osteoarthritis. *Arch Phys Med Rehabil*. 2017;98(6):1210-1216.e1. doi:10.1016/j.apmr.2016.11.024. Epub 2016 Dec 27. PMID: 28034720.	Arthritis

Table S1. List of Included Studies Detailing Formative Research Methods for Attribute Selection and Development

Title	Authors	Year	Objective	Citation	Condition/ Intervention
**A protocol for a discrete choice experiment: understanding patient medicine preferences for managing chronic non-cancer pain**	Marian Shanahan, Briony Larance, Suzanne Nielson, Milton Cohen, Maria Schaffer, Gabrille Campbell	2018	To identify and value the factors that influence important treatment decisions among people living with chronic non-cancer pain (CNCP), so we can better understand the choices they make<br><br>Specifically, we will assess:<br>• Preferences for medicines<br>• Impact on choice of potential side effects including the possibility of addiction<br>• Willingness to pay (WTP) out of pocket for preferred options, and the extent to which costs may be a barrier<br>• The extent to which having input into treatment is important<br>• The degree to which pain interference is tolerated	Shanahan M, Larance B, Nielsen S, Cohen M, Schaffer M, Campbell G. A protocol for a discrete choice experiment: understanding patient medicine preferences for managing chronic non-cancer pain. *BMJ Open*. 2019;9(8):e027153. doi:10.1136/bmjopen-2018-027153. PMID: 31377695; PMCID: PMC6687015.	Chronic non-cancer pain
**Development of a discrete-choice experiment (DCE) to elicit adolescent and parent preferences for hypodontia treatment**	Sophy Barber, Hilary Bekker, Joachim Marti, Sue Pavitt, Balvinder Khambay, David Meads	2018	To develop and test a discrete-choice experiment (DCE) survey to elicit adolescent and parent preferences for dental care for hypodontia	Barber S, Bekker H, Marti J, Pavitt S, Khambay B, Meads D. Development of a discrete-choice experiment (DCE) to elicit adolescent and parent preferences for hypodontia treatment. *Patient*. 2019;12(1):137-148. doi:10.1007/s40271-018-0338-0. PMID: 30367434; PMCID: PMC6335368.	Hypodontia
**A discrete choice experiment on preferences of patients with rheumatoid arthritis regarding disease-modifying antirheumatic drugs: the identification, refinement, and selection of attributes and levels**	Elke Ge Mathijssen, Milou van Heuckelum, Liset van Dijk, Marcia Vervloet, Simone Mt Zonnenberg, Johanna E Vriezekolk, Bart Jf van den Bemt	2018	To comprehensively describe the identification, refinement, and selection of attributes and levels for a discrete choice experiment (DCE) on preferences of patients with rheumatoid arthritis (RA) regarding disease-modifying antirheumatic drugs (DMARDs)	Mathijssen EG, van Heuckelum M, van Dijk L, et al. A discrete choice experiment on preferences of patients with rheumatoid arthritis regarding disease-modifying antirheumatic drugs: the identification, refinement, and selection of attributes and levels. *Patient Prefer Adher*. 2018;12:1537-1555. doi:10.2147/PPA.S170721. PMID: 30197505; PMCID: PMC6112777.	Arthritis
**Perspectives of patients, first-degree relatives and rheumatologists on preventive treatments for rheumatoid arthritis: a qualitative analysis**	Sarah Munro, Luke Spooner, Katherine Milbers, Marie Hudson, Cheryl Koehn, Mark Harrison	2018	• To identify the potential attributes involved in decisions around whether or not to take preventive treatment for RA,<br>• To inform the development of a DCE that would subsequently be used to ascertain the preferences of people at risk of developing RA for development of a preventive treatment program for RA	Munro S, Spooner L, Milbers K, Hudson M, Koehn C, Harrison M. Perspectives of patients, first-degree relatives and rheumatologists on preventive treatments for rheumatoid arthritis: a qualitative analysis. *BMC Rheumatol.* 2018;2:18. doi:10.1186/s41927-018-0026-7. PMID: 30886969; PMCID: PMC6390586.	Arthritis

Table S1. List of Included Studies Detailing Formative Research Methods for Attribute Selection and Development

Title	Authors	Year	Objective	Citation	Condition/ Intervention
**Attitudes toward prenatal screening for chromosomal abnormalities: a focus group study**	Sarah Munro, Julie Sou, Wei Zhang, Tima Mohammadi, Logan Trenaman, Sylvie Langlois, Aslam H Anis	2019	• To address the gap in qualitative reporting of DCE survey development<br>• To provide a case study of the qualitative research process for developing the conceptual attributes for a DCE for prenatal screening and diagnosis<br>• To identify potential attributes, levels, decision-making characteristics, and other factors that may be relevant for designing a DCE to assess preferences for prenatal screening and diagnosis among women and partners/support people in British Columbia	Munro S, Sou J, Zhang W, Mohammadi T, Trenaman L, Langlois S, Anis AH. Attitudes toward prenatal screening for chromosomal abnormalities: a focus group study. *Women Birth*. 2019;32(4):364-371. doi:10.1016/j.wombi.2018.09.006. Epub 2018 Sep 27. PMID: 30270016.	Pregnancy
**A discrete choice experiment on preferences of patients with low back pain about non-surgical treatments: identification, refinement and selection of attributes and levels**	Thomas G Poder, Marion Beffarat, Maria Benkhalti, Ginette Ladouceur, Pierre Dagenais	2019	To comprehensively describe the process of identification, refinement and selection of attributes and levels for a discrete choice experiment (DCE)	Poder TG, Beffarat M, Benkhalti M, Ladouceur G, Dagenais P. A discrete choice experiment on preferences of patients with low back pain about non-surgical treatments: identification, refinement and selection of attributes and levels. *Patient Prefer Adher*. 2019;13:933-940. doi:10.2147/PPA.S201401. PMID: 31354247; PMCID: PMC6576121.	Lower back pain
**Video or in-clinic consultation? Selection of attributes as preparation for a discrete choice experiment among key stakeholders**	Irit Chudner, Margalit Goldfracht, Hadass Goldblatt, Anat Drach-Zahavy, Khaled Karkabi	2019	• To identify relevant attributes and levels of a stakeholder's choice of VC over traditional I-CC for DCE questionnaire development.<br>• To gain insights relevant for appropriate scenario design for a future DCE quantitative stage to be conducted in 3 stakeholder groups in parallel—patients, PCPs and PMs.	Chudner I, Goldfracht M, Goldblatt H, Drach-Zahavy A, Karkabi K. Video or in-clinic consultation? Selection of attributes as preparation for a discrete choice experiment among key stakeholders. *Patient*. 2019;12(1):69-82. doi:10.1007/s40271-018-0318-4. PMID: 29948961.	Video or in-clinic consultation
**Preferences of people with type 2 diabetes for telemedical lifestyle programs in Germany: protocol of a discrete choice experiment**	Jana Sommer, Jan Dyczmons, Sandra Grobosch, Veronika Gontscharuk, Markus Vomhof, Michael Roden, Andrea Icks	2020	• To measure the preferences of people with T2DM regarding telemedical lifestyle programs and coaching approaches and to analyse the heterogeneity of these preferences<br>• To investigate whether preferences predict program success<br>• To compare participants' preferences before and after the intervention	Sommer J, Dyczmons J, Grobosch S, et al. Preferences of people with type 2 diabetes for telemedical lifestyle programs in Germany: protocol of a discrete choice experiment. *BMJ Open*. 2020;10(9):e036995. doi:10.1136/bmjopen-2020-036995. PMID: 32907900; PMCID: PMC7482475.	Type 2 diabetes

Table S1. List of Included Studies Detailing Formative Research Methods for Attribute Selection and Development

Title	Authors	Year	Objective	Citation	Condition/ Intervention
**A discrete choice experiment on women's preferences for water immersion during labor and birth: identification, refinement and selection of attributes and levels**	Thomas G Poder, Nathalie Carrier, Mathieu Roy, Chantal Camden	2020	To use a mixed-method approach to identify attributes and levels that would be included in a discrete choice experiment (DCE) questionnaire to evaluate women's preferences for water immersion during labor and birth	Poder TG, Carrier N, Roy M, Camden C. A discrete choice experiment on women's preferences for water immersion during labor and birth: identification, refinement and selection of attributes and levels. 2020;17(6):1936. doi:10.3390/ ijerph17061936. PMID: 32188019; PMCID: PMC7142518.	Pregnancy
**Eliciting gastric cancer survivors' preferences for follow-up services: a discrete choice experiment protocol**	Hui-qin Li, Jin-hua Han, Hua Yuan, Guang-ying Wan, Hui Xue, Xiu-ying Zhang	2021	• To explore follow-up service-related characteristics that may affect gastric cancer survivors' choices about their follow-up<br>• To elicit how gastric cancer survivors consider the trade-offs among different follow-up service options<br>• To ascertain whether gastric cancer survivors' needs and preferences for follow-up vary due to the economy, politics, technology and culture in different regions	Li HQ, Han JH, Yuan H, Wan GY, Xue H, Zhang XY. Eliciting gastric cancer survivors' preferences for follow-up services: a discrete choice experiment protocol. *BMJ Open*. 2021;11(11):e049742. doi:10.1136/ bmjopen-2021-049742. PMID: 34782340; PMCID: PMC8593722.	Oncology
**Preferences of people with mild cognitive impairment for physical activity interventions in China: protocol for a discrete choice experiment study**	Chang Liu, Hong Yang, Yuchen Jiao, Yunyue Liu, Jing Chang, Yan Ji	2022	• To identify and explore which components of exercise intervention programs for MCI patients are essential<br>• To measure MCI patients' preferences for exercise interventions and summarise relevant characteristics that may influence preference choices<br>• To determine whether these preferences vary by participant characteristics and to classify population types based on the participants' sociodemographic characteristics	Liu C, Yang H, Jiao Y, Liu Y, Chang J, Ji Y. Preferences of people with mild cognitive impairment for physical activity interventions in China: protocol for a discrete choice experiment study. *BMJ Open*. 2022;12(10):e064153. doi:10.1136/bmjopen-2022-064153. PMID: 36241356; PMCID: PMC9577920.	Mild cognitive impairment
**Preferences of oral nutritional supplement therapy among postoperative patients with gastric cancer: attributes development for a discrete choice experiment**	Qiuchen Wang, Yahong Chen, Yi Peng, Hua Yuan, Zhiming Chen, Jia Wang, Hui Xue, Xiuying Zhang	2022	To comprehensively describe the identification, refinement, and selection of attributes and levels for a discrete choice experiment	Wang Q, Chen Y, Peng Y, et al. Preferences of oral nutritional supplement therapy among postoperative patients with gastric cancer: Attributes development for a discrete choice experiment. *PLoS One*. 2022;17(9):e0275209. doi:10.1371/ journal.pone.0275209. PMID: 36174091; PMCID: PMC9522277.	Oncology

Table S1. List of Included Studies Detailing Formative Research Methods for Attribute Selection and Development

Title	Authors	Year	Objective	Citation	Condition/ Intervention
**Qualitative research informing a preference study on selecting cannabis for cancer survivor symptom management: design of a discrete choice experiment**	Colene Bentley, Sara Izadi-Najafabadi, Adam Raymakers, Helen McTaggart-Cowan	2022	Outlines the qualitative research undertaken to design a discrete choice experiment (DCE) aimed at understanding Canadian cancer survivors' preferences for managing their cancer symptoms with cannabis in this complex socio-medical context	Bentley C, Izadi-Najafabadi S, Raymakers A, McTaggart-Cowan H. Qualitative research informing a preference study on selecting cannabis for cancer survivor symptom management: design of a discrete choice experiment. *Patient*. 2022;15(4):497-507. doi:10.1007/s40271-021-00567-3. Epub 2022 Feb 8. PMID: 35132605; PMCID: PMC9197893.	Oncology
**The systematic development of attributes and levels for a discrete choice experiment of HIV patient preferences for long-acting antiretroviral therapies in the United States**	Aaron T Brah, Douglas Barthold, Brett Hauber, Ann C Collier, Rodney J Y Ho, Vincent C Marconi, Jane M Simoni, Susan M Graham	2022	To describe a systematic process used to identify attributes and levels for a discrete choice experiment (DCE) designed to elicit preferences for potential LA-ART options in the US	Brah AT, Barthold D, Hauber B, et al. The systematic development of attributes and levels for a discrete choice experiment of HIV patient preferences for long-acting antiretroviral therapies in the United States. *AIDS Res Ther*. 2022;19(1):13. doi:10.1186/s12981-022-00435-6. PMID: 35216610; PMCID: PMC8881811.	HIV
**Patient and public preferences for coordinated care in Switzerland: development of a discrete choice experiment**	Anna Nicolet, Clémence Perraudin, Joël Wagner, Ingrid Gilles, Nicolas Krucien, Isabelle Peytremann-Bridevaux, Joachin Marti	2022	To develop and test a discrete choice experiment (DCE) eliciting public and patient preferences for better-coordinated care in Switzerland	Nicolet A, Perraudin C, Wagner J, et al. Patient and public preferences for coordinated care in Switzerland: development of a discrete choice experiment. *Patient*. 2022;15(4):485-496. doi:10.1007/s40271-021-00568-2. Epub 2022 Jan 24. PMID: 35067858; PMCID: PMC9197802.	Coordinated care
**Identifying patient preferences for diabetes care: a protocol for implementing a discrete choice experiment in Samoa**	Anna C Rivara, Omar Galárraga, Melania Selu, Maria Arorae, Ruiyan Wang, Kima Faasalele-Savusa, Rochelle Rosen, Nicola L Hawley, Satupaitea Viali	2023	• To design, pilot, and implement a DCE measuring diabetes care preferences in Samoa<br>• To identify and measure the strength of preference and importance of attributes of diabetes care alternatives and standard care options<br>• To identify the trade-offs (i.e., marginal Willingness to Pay (WTP)), participants are willing to make to obtain preferred attributes, and the probability of uptake for such services<br>• To measure longitudinal changes in care preferences over time	Rivara AC, Galárraga O, Selu M, et al. Identifying patient preferences for diabetes care: a protocol for implementing a discrete choice experiment in Samoa. *PLoS One*. 2023;18(12):e0295845. doi:10.1371/journal.pone.0295845. PMID: 38134044; PMCID: PMC10745180.	Diabetes

Table S1. List of Included Studies Detailing Formative Research Methods for Attribute Selection and Development

Title	Authors	Year	Objective	Citation	Condition/ Intervention
**Eliciting preferences of persons with dementia and informal caregivers to support ageing in place in the Netherlands: a protocol for a discrete choice experiment**	Isabelle Vullings, Joost Wammes, Özgül Uysal-Bozkir, Carolien Smits, Nanon H M Labrie, J D Swait, Esther de Bekker-Grob, Janet L Macneil-Vroomen	2023	To identify individual and joint preferences of persons with dementia and informal caregivers for in-home support that enables AIP.<br>To create optimal healthcare packages for persons with dementia and informal caregivers to AIP. For this second aim, choice model inferences will be the basis for creating the most preferred care packages and testing the uptake in a subsample of participants. This will validate the national model inferences and provide policy makers with high quality, understandable and implementable evidence.	Vullings I, Wammes J, Uysal-Bozkir Ö, et al. Eliciting preferences of persons with dementia and informal caregivers to support ageing in place in the Netherlands: a protocol for a discrete choice experiment. *BMJ Open*. 2023;13(12):e075671. doi:10.1136/bmjopen-2023-075671. PMID: 38072475; PMCID: PMC10729270.	Dementia
**A discrete choice experiment to elicit preferences for a liver screening program in Queensland, Australia: a mixed methods study to select attributes and levels**	Michelle J Allen, Rachael Doran, David Brain, Elizabeth E Powell, James O'Beirne, Patricia C Valery, Adrian Barnett, Ruvini Hettiarachchi, Ingrid J Hickman, & Sanjeewa Kularatna	2023	To create a list of potential attributes and levels which can be used in a DCE study to elicit preferences for chronic liver disease screening programs.	Allen, M.J., Doran, R., Brain, D. et al. A discrete choice experiment to elicit preferences for a liver screening program in Queensland, Australia: a mixed methods study to select attributes and levels. *BMC Health Serv Res.* 2023;23, 950. doi:10.1186/s12913-023-09934-2	Chronic Liver disease
**Development of a discrete choice experiment questionnaire to elicit preferences by pregnant women and policymakers for the expansion of non-invasive prenatal screening**	Hung Manh Nguyen, Carmen Lindsay, Mohammad Baradaran, Jason Robert Guertin, Leon Nshimyumukiza, Bounhome Soukkhaphone, Daniel Reinharz	2023	This study aimed to develop a discrete choice experiments (DCE) questionnaire to elicit the preferences of patients and policymakers. The instrument was specifically developed to estimate preferences for new conditions to be added to a screening program for fetal chromosomal anomalies.	Nguyen HM, Lindsay C, Baradaran M, et al. Development of a discrete choice experiment questionnaire to elicit preferences by pregnant women and policymakers for the expansion of non-invasive prenatal screening. *PLoS One*. 2023;18(6):e0287653. doi:10.1371/journal.pone.0287653. PMID: 37352239; PMCID: PMC10289448.	Pregnancy

Table S1. List of Included Studies Detailing Formative Research Methods for Attribute Selection and Development

Title	Authors	Year	Objective	Citation	Condition/ Intervention
**Patient and public acceptance of digital technologies in health care: protocol for a discrete choice experiment**	Ann-Kathrin Fischer, Axel C Mühlbacher	2023	Our primary objective is to weight the criteria that impact patient and public acceptance.<br><br>Secondary objectives include:<br>• A benefit-burden assessment (estimation of the maximum acceptable burden of technical features and therapy-related characteristics for the patient or individual, eg, no human contact)<br>• Overall comparison (assessment of the relative importance of attributes for comparing digital technologies)<br>• Adherence (identification of key attributes that influence patient adherence)<br><br>Our exploratory objectives include:<br>• Heterogeneity assessment (how preferences differed based on participant characteristics)<br>• Subgroup analysis (to explore and explain heterogeneity based on the correlation between therapy preferences and participants' sociodemographic information, experience-based treatment history, and attitudes).<br>• Our methodological aims are to investigate the use of DCE and the impact on data quality, preferences, and choice consistency.	Fischer AK, Mühlbacher AC. Patient and public acceptance of digital technologies in health care: protocol for a discrete choice experiment. *JMIR Res Protoc*. 2023;12:e46056. doi:10.2196/46056. PMID: 37561559; PMCID: PMC10450540.	Digital technologies in healthcare
**Eliciting depression patients' preferences for medication management: a protocol for discrete choice experiment**	Peng Xie, Hui-Qin Li, Wan-Lin Peng, Hao Yang	2024	To outline an ongoing DCE that aims to:<br>• Explore medication-management-related characteristics that may affect depression patients' adherence to antidepressant<br>• Elicit how depression patients consider the trade-offs among different medication managements	Xie P, Li HQ, Peng WL, Yang H. Eliciting depression patients' preferences for medication management: a protocol for discrete choice experiment. 2024;18:289-300. doi:10.2147/PPA.S444800. PMID: 38327728; PMCID: PMC10849879.	Depression

Table S1. List of Included Studies Detailing Formative Research Methods for Attribute Selection and Development

Title	Authors	Year	Objective	Citation	Condition/ Intervention
**Developing a discrete choice experiment instrument for evaluating patients' preferences in precision oncology**	Zahra Karimi Majd, Nazila Yousefi, Mohammad Peikanpour, Mohammad Sistanizad, Ghader Mohammadnezhad, Behniya Azadmehr, Farzad Peiravian	2024	• To identify and prioritize attributes of precision oncology that are important for patients to develop and validate a standard stated preference instrument<br>• To seek consensus from experts in Iran regarding the attributes of precision oncology to inform subsequent research and develop a standard and valid DCE instrument to measure patient preferences, as researchers developing a DCE are tasked with creating a limited set of attributes based on a rigorous process, including a literature review and expert opinions to make sure the DCE instrument is standard and not overly complex or burdensome	Karimi Majd Z, Yousefi N, Peikanpour M, et al. Developing a discrete choice experiment instrument for evaluating patients' preferences in precision oncology. *Iran J Pharm Res*. 2024;22(1):e141797. doi:10.5812/ijpr-141797.	Oncology
